# Double vitrification and warming does not compromise the chance of live birth after single unbiopsied blastocyst transfer

**DOI:** 10.1093/hropen/hoad037

**Published:** 2023-09-22

**Authors:** S Makieva, M K Sachs, M Xie, A Velasco, S El-Hadad, D R Kalaitzopoulos, I Dedes, R Stiller, B Leeners

**Affiliations:** Department of Reproductive Endocrinology, University Hospital Zurich, Zurich, Switzerland; Department of Reproductive Endocrinology, University Hospital Zurich, Zurich, Switzerland; Department of Reproductive Endocrinology, University Hospital Zurich, Zurich, Switzerland; Department of Reproductive Endocrinology, University Hospital Zurich, Zurich, Switzerland; Department of Reproductive Endocrinology, University Hospital Zurich, Zurich, Switzerland; Department of Gynaecology, University Hospital Zurich, Zurich, Switzerland; Department of Reproductive Endocrinology, University Hospital Zurich, Zurich, Switzerland; Department of Gynaecology, University Hospital Zurich, Zurich, Switzerland; Department of Gynaecology, University Hospital Zurich, Zurich, Switzerland; Department of Reproductive Endocrinology, University Hospital Zurich, Zurich, Switzerland; Department of Reproductive Endocrinology, University Hospital Zurich, Zurich, Switzerland

**Keywords:** vitrification, birth, pregnancy, blastocyst, double vitrification, cryopreservation

## Abstract

**STUDY QUESTION:**

Does double vitrification and thawing of an embryo compromise the chance of live birth after a single blastocyst transfer?

**SUMMARY ANSWER:**

The live birth rate (LBR) obtained after double vitrification was comparable to that obtained after single vitrification.

**WHAT IS KNOWN ALREADY:**

Double vitrification-warming (DVW) is commonly practiced to accommodate surplus viable embryos suitable for transfer, to allow retesting of inconclusively diagnosed blastocysts in preimplantation genetic testing (PGT), and to circumvent limitations associated with national policies on embryo culture in certain countries. Despite its popularity, the evidence concerning the impact of DVW practice on ART outcomes is limited and lacking credibility. This is the first thorough investigation of clinical pregnancy and LBR following DVW in the case where the first round of vitrification occurred at the zygote stage and the second round occurred at the blastocyst stage in the absence of biopsy.

**STUDY DESIGN, SIZE, DURATION:**

This is a retrospective observational analysis of n = 407 single blastocyst transfers whereby embryos created by IVF/ICSI were vitrified-warmed once (single vitrification-warming (SVW) n = 310) or twice (DVW, n = 97) between January 2017 and December 2021.

**PARTICIPANTS/MATERIALS, SETTING, METHODS:**

In the SVW group, blastocysts were vitrified on Day 5/6 and warmed on the day of embryo transfer (ET). In the DVW group, two pronuclear (2PN) zygotes were first vitrified-warmed and then re-vitrified on Day 5/6 and warmed on the day of ET. Exclusion criteria were ETs from PGT and vitrified-warmed oocyte cycles. All of the ETs were single blastocyst transfers performed at the University Hospital Zurich in Switzerland following natural or artificial endometrial preparation.

**MAIN RESULTS AND THE ROLE OF CHANCE:**

The biochemical pregnancy rate, clinical pregnancy rate (CPR), and LBR were all comparable between the DVW and SVW groups. The CPR for DVW was 44.3% and for SVW it was 42.3% (*P* = 0.719). The LBR for DVW was 30.9% and for SVW it was 28.7% (*P* = 0.675). The miscarriage rate was additionally similar between the groups: 27.9% for DVW and 32.1% for SVW groups (*P* = 0.765).

**LIMITATIONS, REASONS FOR CAUTION:**

The study is limited by its retrospective nature. Caution should be taken concerning interpretation of these findings in cases where DVW occurs at different stages of embryo development.

**WIDER IMPLICATIONS OF THE FINDINGS:**

The result of the present study on DVW procedure provides a framework for counselling couples on their chance of clinical pregnancy per warming cycle. It additionally provides confidence and reassurance to laboratory professionals in certain countries where national policies limit embryo culture strategies making DVW inevitable.

**STUDY FUNDING/COMPETING INTEREST(S):**

This work was supported by the University Research Priority Program ‘Human Reproduction Reloaded’ of the University of Zurich. The authors have no conflict of interest related to this study to declare.

**TRIAL REGISTRATION NUMBER:**

N/A.

WHAT DOES THIS MEAN FOR PATIENTS? The ability to freeze surplus embryos has revolutionized modern assisted reproductive technologies and improved the fertility care of subfertile patients, giving the option to perform more embryo transfers from a single IVF cycle. Embryos can be frozen at various stages of development, including the zygote (early) and blastocyst (late) stages, and the transfer of frozen-warmed embryos has been shown to result in comparable clinical outcomes to fresh embryo transfer. Occasionally patients are faced with the information that their embryos have undergone more than one round of freezing and warming procedure but the evidence surrounding the impact of repeated cryopreservation on the chance of pregnancy and live birth is limited. Our work reassures both clinicians and patients that embryos that have been frozen twice, first as zygotes and thereafter as blastocysts, have equal chances of resulting in a live birth when transferred in the womb, compared with embryos that have been frozen only once. Patients should feel confident when transferring embryos that have undergone more than one cryopreservation procedure.

## Introduction

Frozen embryo transfer (FET) has undoubtedly revolutionized modern ART practice. Indeed, embryo cryopreservation has re-defined and increased the cumulative live birth rate (LBR), allowed for deferred embryo transfer (ET) in cases of medical indications, popularized single embryo transfer (SET), and facilitated preimplantation diagnostics (PGT). Advancement of embryo cryopreservation methodology has helped equalize the chance of live birth after FET and fresh cycles (Roque *et al.*, 2019; Zaat *et al.*, 2021). Consequently, the statistics associated with FETs are trending upwards, including freeze-all cases (where all fresh embryos are cryopreserved) as well as the number of surplus embryos from FET cycles which are destined to be re-cryopreserved. Repeated cryopreservation has the potential to further increase the cumulative clinical pregnancy rate (CPR), reduce the risk of multiple pregnancies, allow for re-biopsy in the case of inconclusive PGT diagnosis, and decrease the cumulative cost of IVF treatment. To date, only a handful of studies have explored the effect that two cryopreservation procedures have on the same embryo, in the absence of biopsy, with regards to clinical outcomes. Among these studies, the inclusion of cohorts of embryos frozen at various embryonic stages and the use of various cryopreservation protocols within each group has limited interpretation of the results (Kumasako *et al.*, 2009; Koch *et al.*, 2011; Murakami *et al.*, 2011; Zheng *et al.*, 2017; Farhi *et al.*, 2019). The most recent multicentre study employing a cohort of vitrified and slow-frozen embryos in a retrospective analysis showed no detrimental impact of double cryoconservation on clinical and neonatal outcomes (Hallamaa *et al.*, 2021). The competence of biopsied euploid blastocysts after two rounds of cryopreservation has also been explored with contradictory results (Taylor *et al.*, 2014; Bradley *et al.*, 2017; Cimadomo *et al.*, 2018; Aluko *et al.*, 2021). Strikingly, none of the aforementioned studies have explored the chance of live birth after double vitrification-warming (DVW) when the first round of vitrification has occurred at the two pronuclear (2PN) zygote stage. It is noteworthy that cryopreservation of 2PN zygotes is a pivotal part of routine IVF laboratory practice in countries where embryo culture is regulated by national policies. For example, in Germany and Switzerland, only a limited number of zygotes are allowed to remain in culture, with all remaining 2PN zygotes being destined to cryopreservation. What is more, numerous IVF centres worldwide prefer vitrifying 2PN zygotes than cleavage stage embryos due to a number of advantages associated with the former approach (Troup *et al.*, 1991; Senn *et al.*, 2000). Hence, whether the practice of DVW involving 2PN zygotes and blastocysts hinders clinical outcomes is currently still an open question. With recent emerging evidence that FET might not be an innocent strategy when perinatal and maternal outcomes are concerned, we should be opting to at least securing confidence in the methodology of the cryopreservation protocol (Sargisian *et al.*, 2022). Herein, we address thoroughly for the first time the transfer outcomes of unbiopsied embryos vitrified twice, first as a 2PN zygote and then as a blastocyst.

## Materials and methods

### Study design

**Figure 1.**
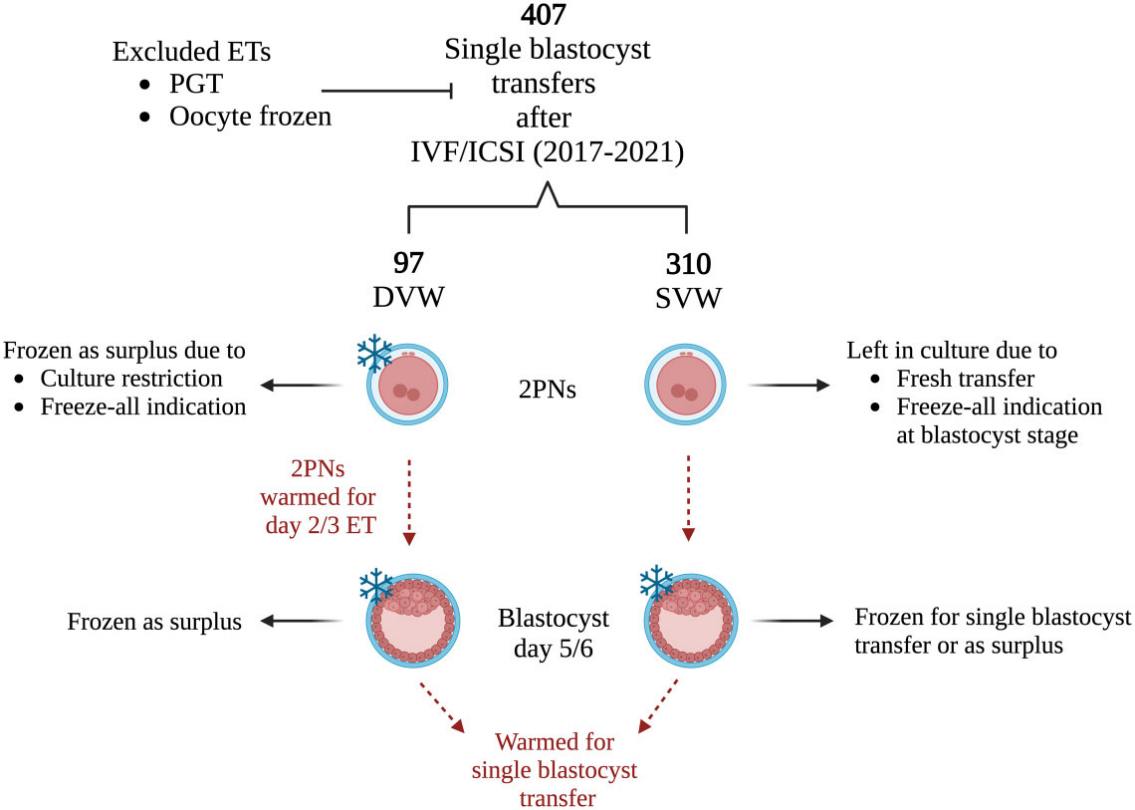
**Illustration of the study design.** The indications for vitrification at each embryo stage are indicated with arrows. DVW: double vitrification warming; SVW: single vitrification warming; 2PN: two pronuclear stage zygotes; ET: embryo transfer; PGT: preimplantation genetic testing. Figure created with BioRender.com.

This is a retrospective observational analysis of 407 single blastocyst transfers from frozen-thawed cycles whereby the embryo was either subjected to vitrification once (single vitrification-warming (SVW), n = 310) or twice (DVW, n = 97) between January 2017 and December 2021 at the University Hospital Zurich in Switzerland. In the SVW group, the blastocysts were vitrified on Day 5/6 and warmed on the day of ET. In the DVW group, the zygotes at 2PN stage were first vitrified-warmed and then re-vitrified on Day 5/6 and warmed on the day of ET. Exclusion criteria were ETs from PGT cycles and vitrified-warmed oocyte cycles. Subjects had given their written informed consent for their data to be used for research and the local ethical committee on human research had approved the research protocol. The indications for vitrification included the freeze-all strategy to circumvent ovarian hyperstimulation syndrome (OHSS) or banking surplus embryos due to either embryo culture restriction laws or SET. All ETs were performed following a natural or artificial endometrial preparation for a single blastocyst transfer. The study design is illustrated in Figure 1. Characteristics of the cycles included and the patients belonging to the two cohorts are presented in Tables 1 and 2, respectively. All ETs included in this study belonged either to the SVW or the DVW group and there was no patient with ET in both groups.

**Table 1.** Characteristics of the 272 cycles included.

Cycle characteristics	DVW	SVW	*P* value
**Number of cycles**	76	196	
**Maternal age at oocyte retrieval (years)**	35.06 ± 4.2	35.7 ± 4.3	0.237
**Cause of infertility**			0.988
Idiopathic	14/76 (5.2%)	37/196 (18.8%)	
Anovulation	3/76 (3.9%)	12/196 (6.1%)	
Endometriosis	7/76 (9.2%)	17/196 (8.6%)	
PCOS	5/76 (6.5%)	13/196 (6.6%)	
Male factor	31/76 (40.7%)	78/196 (39.7%)	
Genetic	2/76 (2.6%)	5/196 (2.5%)	
Others	5/76 (6.5%)	8/196 (4.0%)	
Multiple	9/76 (11.8%)	26/196 (13.2%)	
**Stimulation protocol of fresh cycle**			0.412
Short	33/76 (43.4%)	71/196 (36.2%)	
Long	13/76 (17.1%)	46/196 (23.4%)	
Antagonist	30/76 (3.9%)	79/196 (40.3%)	
**Sperm source**			
Fresh	65/76 (85.5%)	174/196 (88.7%)	0.762
Frozen	6/76 (7.8%)	12/196 (6.1%)	
TESE	5/76 (6.5%)	10/196 (5.1%)	
**Mean number of oocytes recovered**			
Overall	17.19 ± 8.5	15.29 ± 8.5	0.323
MII	10.19 ± 7.4	8.58 ± 7.4	0.351
MI	1.0 ± 1.4	1.0 ± 1.4	0.713
GV	2.0 ± 2.4	1.79 ± 2.4	0.999
**Mean fertilisation rate (%)**	74.5 ± 16.8	74.0 ± 17.0	0.853
**Blastocyst cryosurvival rate (%)**	98.4 ± 8.5	98.7 ± 9.0	0.26
**2PN cryosurvival rate (%)**	97.9 ± 9.2	na	
**Blastocyst formation rate^a^ (%)**	35.02 ± 12.21	35.1 ± 12.27	0.494
**Blastocyst formation rate^b^ (%)**	66.04 ± 24.88	67.01 ± 24.90	0.798
**Frozen ETs per ovarian stimulation cycle**			0.063
1	58/76 (76.3%)	131/196 (66.8%)	
2	16/76 (21%)	40/196 (20.4%)	
3	02/76 (2.6%)	13/196 (6.6%)	
≥4	0/76 (0%)	12/197 (6.1%)	

Data presented as n (%) or mean (±SD: standard deviation) and analysed using chi-square, Fisher Exact, or Mann–Whitney U-test.

DVW: double vitrification-warming; SVW: single vitrification-warming; ET: embryo transfer;  TESE: testicular sperm extraction; MII: metaphase II; MI: metaphase I; GV: germinal vesicle; PN: bipronuclear zygotes.

^a^ Blastocyst formation rate per zygotes thawed.

^b^ Blastocyst formation rate per zygotes left in extended culture.

**Table 2.** Embryo transfer characteristics of DVW and SVW cohorts.

Embryo transfer characteristics	DVW	SVW	*P* value
**Number of ETs**	97	310	
**Mean maternal age ET (years)**	36.65 ± 4.1	36.61 ± 4.2	0.909
**Fertilisation method of transferred blastocyst**			0.926
IVF	28/97 (28.8%)	91/310 (29.3%)	
ICSI	69/97 (71.13%)	219/310 (70.6%)	
**Blastocyst quality at ET**			0.825
Top (AA)	32/97 (32.9%)	112/310 (36.1%)	
Good (AB, BA)	30/97 (30.9%)	95/310 (30.6%)	
Medium (BB)	35/97 (36.0%)	103/310 (33.2%)	
**Mean endometrial thickness (mm)**	8.47 ± 1.7	8.48 ± 1.8	0.812
**Endometrial preparation**			0.056
Spontaneous	2/97 (2.0%)	0/310 (0.0%)	
Artificial	95/97 (98%)	310/310 (100%)	
**Blastocyst quality at cryopreservation**			0.985
Top (AA)	33/97 (34.0%)	108/310 (34.8%)	
Good (AB, BA)	30/97 (30.9%)	96/310 (30.9%)	
Medium (BB)	34/97 (35.0%)	106/310 (34.1%)	
**Blastocyst expansion score at cryopreservation**			0.696
6	1/97 (1.0%)	0/310 (0.0%)	
5	4/97 (4.1%)	16/310 (5.1%)	
4	72/97 (74.2%)	224/310 (72.2%)	
2 or 3	20/97 (20.6%)	70/310 (22.5%)	
**Time interval between oocyte retrieval and ET**			**<0.001**
Within 3 months	0/97 (0.0%)	111/310 (35.8%)	
Within 4–6 months	18/97 (18.5%)	79/310 (25.4%)	
Within 7–12 months	31/97 (31.9%)	66/310 (21.2%)	
Within 13–24 months	20/97 (20.6%)	30/310 (9.6%)	
After 2 years	28/97 (28.8%)	24/310 (7.7%)	

Data presented as n (%) or mean (±SD: standard deviation) and analysed using chi-square, Fisher Exact, or Mann–Whitney U-test.

Blastocyst quality: first letter denotes quality of ICM and second letter quality of TE according to Gardner’s criteria.

DVW: double vitrification-warming; SVW: single vitrification-warming; ET: embryo transfer;  C: cryopreservation.

### Ovarian stimulation

Prior stimulation women received a gestagen (10 mg/day) for 10 days up to 28 days, beginning at the second cycle day, in the short or antagonist protocols, and a GnRH-agonist (triptorelin, 0.1 mg/day) on cycle Day 21 for the long protocol, of ovarian stimulation (ovarian stimulation). For ovarian stimulation, either a short or long GnRH-agonist protocol or a GnRH-antagonist protocol were used with either application of hMG or recombinant FSH. When at least three follicles with a diameter of ≥17 mm were observed during vaginal ultrasound, final oocyte maturation was induced with either 6500IE hCG in the short or long protocols or with the addition of a GnRH-agonist accompanied by about 1600 IE hCG in the GnRH-antagonist protocol. Ultrasound guided oocyte retrieval was performed about 36 h after administration of the hCG/GnRH-agonist trigger. For artificial endometrial preparation, oral estrogen at 6 mg/days followed by the addition of vaginal progesterone at 1000 mg/day was initiated 5 days prior to blastocyst transfer.

### Laboratory procedures

The follicular fluid was collected in preheated round bottom 14 ml tubes held in a heating block calibrated at 37°C. The oocyte search was done under laminar flow using 60 mm flat bottom petri dishes. All cumulus–oocyte complexes (COCs) were cultured in a humidified incubator with conditions of 37°C and 6% CO_2_ in fertilization media (Global for Fertilisation, CooperSurgical, Trumbull, Connecticut, USA or G-IVF Vitrolife, Gothenburg, Sweden) under oil overlay (OVOIL, Vitrolife). Insemination was performed 5–7 h after oocyte retrieval by means of IVF or ICSI depending predominantly on the semen parameters. For ICSI, oocytes were denuded using hyaluronidase enzyme (80 IU/ml) and, following insemination, were incubated in Global Total or G1 Vitrolife media in a microdroplet dish (Vitrolife) in an incubator with conditions of 6% CO_2_, 5% O_2_ and 37°C. For IVF, 100 000 motile spermatozoa/oocyte were used to inseminate COCs distributed in the wells of 4-well dishes in 700 µl of G-IVF or Global for Fertilisation media under oil overlay. At 16–19 h after insemination, oocytes were inspected for the presence of 2PNs and two polar bodies. Only two to three 2PN zygotes randomly selected for immediate ET were left in culture. Surplus 2PN zygotes were subjected to vitrification for future thawing cycles. Following a fresh ET, surplus blastocysts were frozen on Day 5 or 6 for another thawing cycle. All embryos were frozen by means of vitrification. For the single ETs, blastocysts were cultured in Global Total or G2 Vitrolife media and transferred intrauterine with the Guardia™ Access Embryo Transfer Catheter (COOK Medical, Bloomington, IN, USA) within 1 min of embryo loading.

### Vitrification and warming procedures

The presence of clear 2PN zygotes was confirmed prior to the start of vitrification to avoid vitrifying zygotes in syngamy. The Cryotop vitrification method was applied for all 2PN zygotes and blastocysts cryopreserved. In this method, an open vitrification carrier, which contains a polypropylene strip accompanied by a protective cover, is used. By aspirating the excess solution, that is placed on the filmstrip, only a thin layer covering the cryopreserved cells ultimately remains. By using this minimal volume, the cooling rate is increased up to 2300°C/min and the warming rate is increased up to 4210°C/min (Kuwayama, 2007). The Cryotop method shows a high efficiency for the vitrification, causing only minimal cryodamage. The Kitazato (Kitazato Corporation, Shizuoka, Japan) vitrification (VT601) and thawing (VT602) solutions and the Cryotop^®^ Oocyte/Embryo Vitrification Device Open System were used for this study. No more than three zygotes at the 2PN stage were loaded per cryotop and thawed for ET. The 2PN zygotes were cryopreserved between 17 and 21 h post insemination. No more than one blastocyst was loaded per Cryotop straw and thawed for ET. Blastocysts were graded according to Gardner morphology (Alpha Scientists in Reproductive Medicine and ESHRE Special Interest Group of Embryology, 2011) and artificially collapsed including zona opening (14 µm hole) with a laser prior to cryopreservation when they noted as being expansion grade 4. For expansion grades <4, the blastocysts were vitrified without artificial collapse. Therefore, blastocyst expansion at the time of ET is not reported, as it is biased by the practise of assisted hatching. A grade of at least 3BB on Day 5 and 4BB on Day 6 was acceptable for freezing. Blastocyst of grade C were not vitrified. Following blastocyst warming, the blastocysts were left for at least 2 h to recover prior to the commencement of the ET. Blastocysts previously vitrified as zygotes and re-vitrified without artificial collapse and zona opening, were exposed to zona opening post warming. Cryosurvival rates were calculated per cycle as per the consensus definitions (Alpha Scientists In Reproductive Medicine, 2012; ESHRE Special Interest Group of Embryology and Alpha Scientists in Reproductive Medicine, 2017).

### Outcome measures

The primary outcome analysed was live birth per ET, defined by the birth of one live born infant. The secondary outcomes assessed were: clinical pregnancy (pregnancy visible with ultrasound at a minimum of 8 gestational weeks), biochemical pregnancy (β-hCG level lower than 100 mlU/ml 14 days after ET and a rapid fall in serum β-hCG concentration), and miscarriage (loss of a detected clinical pregnancy up to 22 gestational weeks). The mode of delivery was also assessed in the DVW and SVW groups.

### Statistical analysis

Demographic and clinical variables were analysed using standard statistical tests: nominal variables were compared by Chi-Square test and serial variables were tested for normal distribution using graphical measures and Kolmogorov–Smirnov/Shapiro–Wilk test followed by independent t-test or chi-square test as appropriate. Data were presented as mean (±SD) or n (%). A *P*-value <0.05 was accepted as significant. The statistical analyses and data processing was conducted with SPSS (version 24, IBM, Chicago, IL, USA).

## Results

### Embryo transfer characteristics

We retrospectively identified 310 ETs associated with the SVW strategy and 97 ETs associated with the DVW strategy. The 407 ETs in this study originated from a total of 272 cycles, of which the characteristics were homogenous across the SVW and DVW groups (Table 1). The two groups were also homogeneous for the most ET characteristics examined and considered important for the intended comparison (Table 2). The only ET characteristic that differed between the two groups was the time interval from oocyte retrieval to ET (*P* < 0.001). As expected, opposing trends were observed between the two groups with regards to the time interval from oocyte retrieval to ET, with the SVW group having the largest group of ETs (35.8%) performed within 3 months from oocyte retrieval, while the DVW group had the largest group of ETs performed between 7 and 12 months from oocyte retrieval (31.9%). In the DVW group, as many as 28.8% of ETs were performed after 2 years from oocyte retrieval, while only 7.7% of ETs were performed after the same timeframe in the SVW group. However, this difference was not reflected in the mean maternal age at ET, which was comparable between the two groups.

### Clinical outcomes

All clinical outcomes were comparable between the two groups (Table 3). Specifically, the biochemical pregnancy rate was 17.5% in the DVW group and 13.8% in the SVW group but the 3.7% difference was not statistically significant (*P* = 0.376). The CPR was also comparable between the groups; for DVW it was 44.3% and for SVW it was 42.3%. The miscarriage rate, as a proportion of clinical pregnancies, was 27.9% for women in the DVW group and 32.1% for women in the SVW group. The LBR was 30.9% and 28.7% for the DVW and SVW groups, respectively (*P* = 0.675). The mode of delivery did not differ between the two groups; the majority of women received a caesarean section.

**Table 3.** Clinical outcomes of DVW and SVW.

Outcomes per ET	DVW	SVW	*P* value
**Number of ETs**	97	310	
**Biochemical pregnancy**			0.376
Yes	17 (17.5%)	43 (13.8%)	
No	80 (82.5%)	267 (86.2%)	
**Clinical pregnancy**			0.719
Yes	43 (44.3%)	131 (42.3%)	
No	54 (55.7%)	179 (57.7%)	
**Miscarriage**			0.609
Yes	12 (27.9%)	42 (32.1%)	
No	31 (72.1%)	89 (67.9%)	
**Live birth**			0.675
Yes	30 (30.9%)	89 (28.7%)	
No	67 (69.1%)	221 (71.3%)	
**Mode of delivery**			0.240
Spontaneous	13 (43.3%)	23 (25.8%)	
CS	15 (50.0%)	56 (62.9%)	
Vacuum	3 (10.0%)	10 (11.2%)	

Data presented as n (%) and analysed using chi-square test.

DVW: double vitrification-warming; SVW: single vitrification-warming; ET: embryo transfer; CS: Caesarean section.

## Discussion

This is the first study that explored the impact of two vitrification procedures performed at the zygote and at the blastocyst stage on clinical outcomes after single blastocyst transfers. Our retrospective analysis, comprised of a neat cohort and appropriate design, reassures us that double vitrification is a safe strategy for banking surplus embryos. Indeed, the CPR (44.3%) and LBR (30.9%) after transfer of a warmed blastocyst, which was previously vitrified and warmed at the zygote stage, are all comparable to the control group and in accordance with benchmark values for single ET (ESHRE Special Interest Group of Embryology and Alpha Scientists in Reproductive Medicine, 2017; ESHRE Clinic PI Working Group *et al.*, 2021). Furthermore, the cryosurvival rates after warming that we report for zygotes and blastocysts fall well within the competency and benchmark values (ESHRE Special Interest Group of Embryology and Alpha Scientists in Reproductive Medicine, 2017; Alpha Scientists In Reproductive Medicine, 2012). In addition, our miscarriage rates that are around 30% for both groups fall within the minimally expected competence value (≤35%) set recently by Italian Societies’ KPIs for women over the age of 35 (Vaiarelli *et al.*, 2023). The need for our study emerged from the limited evidence surrounding clinical outcomes of embryos that have endured two rounds of freezing.

We identified 11 studies that have attempted to understand whether cryopreserving and warming embryos twice could negatively influence clinical and neonatal outcomes. The study of Kumasako *et al*. from Japan, is the only study directly comparable to ours as it includes only cases where vitrification was used for cryopreservation (Kumasako *et al.*, 2009). Similar to us, they compared outcomes from one round of vitrification (n = 301) whereby 2PN zygotes were vitrified and thawed and transferred at Day 3 to outcomes from two rounds of vitrification (n = 50) whereby 2PN zygotes were thawed and re-vitrified at the morula or blastocyst stage before being thawed for Day 5 transfer. They reported no difference between the two groups but several limitations with that study should be discussed. Firstly, they do not report the LBR, while their implantation and CPRs are low; 25–28% and 19–25%, respectively in both groups. Secondly, the transfer strategy of multiple embryos at once, and at different developmental days per group (Days 3 and 5), complicates interpretation. Thirdly, all embryos were produced from oocytes of women who experienced OHSS, limiting the importance of the findings to that category of patients. Finally, the patient characteristics as well as the main data were analysed per cycle instead of per ET, which does not answer the question of whether the transfer outcomes of a double or single vitrified embryo are comparable. Our analysis includes only single blastocyst transfers from warming of blastocysts that were either vitrified once as a blastocyst or twice as a blastocyst with a pre-vitrification step at the 2PN stage. The design of our study is not only more homogeneous but also more relevant, as single blastocyst transfer is the gold standard of ET and 2PN vitrification is highly practiced in a plethora of countries restricted by embryo culture laws (De Neubourg *et al.*, 2022).

Three more studies included 2PN zygotes in their cohorts. Murakami and colleagues evaluated live births and perinatal outcomes in twice frozen embryos (n = 92) (Murakami *et al.*, 2011). However, the results from that study are very challenging to interpret due to the inclusion of mixed embryonic stages per group (2PN zygotes, cleavage stage embryos and blastocysts), and cryopreservation (slow freezing and vitrification), as well as the lack of a SET policy and the inclusion of patients who belonged to both groups. Nevertheless, that study also concluded that a similar live birth can be achieved whether an embryo is frozen once and twice before ET, without neonatal complications in either group. In line, a study from Australia that retrospectively compared 55 double and 40 single cryopreserved embryos at different developmental stages (2PN, cleavage and blastocyst stages) found no difference in terms of CPR and LBR (Koch *et al.*, 2011). Notably the LBR was only between 13% and 15% in their population, despite the utilization of a multiple ET strategy, and they also pooled cases processed with slow freezing and vitrification protocols. Recently a two-centre retrospective analysis of single (n = 304) and double (n = 89) cryopreserved embryos with slow freezing and vitrification at variable embryonic stages, including 2PN zygotes, was conducted (Hallamaa *et al.*, 2021). They reported comparable outcomes from both strategies and endorse double cryopreservation for surplus embryos coming from a freeze-thaw cycle.

Surprisingly, both studies which have been conducted to address double vitrification at the cleavage and blastocyst stage, have report compromised clinical outcomes. A retrospective analysis from China employed a combination of slow freezing and vitrification when cryopreserving embryos at the cleavage and blastocyst stage (Zheng *et al.*, 2017). In their study, double vitrification of 127 embryos resulted in a 10% lower LBR. In an attempt to explain an underlying mechanism, the authors argued that the cryopreservation procedures could have elicited transcriptional changes in the embryos enough to hinder their developmental competence in utero. With this regard, only one study has assessed and shown that re-vitrification can alter the RNA of human blastocysts (Daneshvar *et al.*, 2021). Similarly, Farhi and colleagues reported lower pregnancy rates after double cryopreservation (n = 25) with slow freezing at the cleavage and blastocyst stages compared with after single cryopreservation (n = 50) at blastocyst stage only (Farhi *et al.*, 2019).

Finally, five studies on double vitrification have been conducted in the context of embryo biopsy. Taylor and colleagues employed vitrification and slow freezing to cryopreserve once (n = 85) or twice (n = 17) euploid blastocysts and found both strategies to produce comparable clinical outcomes after ET, including a LBR of 50% in the double cryopreserved group (Taylor *et al.*, 2014). A large study from Australia found that biopsied blastocysts vitrified once (n = 2130) or twice (n = 34) have identical clinical outcomes with the latter group recording a 38.5% LBR (Bradley *et al.*, 2017). These findings were confirmed by an Italian multicentre retrospective study that found no difference in clinical pregnancy and LBRs between once-vitrified euploid blastocysts (n = 2825) and twice-vitrified euploid blastocysts (n = 49) after single biopsies (Cimadomo *et al.*, 2018). Two studies, however, showed contrary results. The LBR was strikingly low after re-vitrification of once-biopsied euploid blastocysts (n = 95, LBR = 28.4%) while it was almost double in the once-biopsied once-vitrified group (n = 2603, LBR = 55.1%) (Aluko *et al.*, 2021). A study from China demonstrated higher pregnancy loss and diminished LBRs without perinatal implications in n = 30 cases where blastocysts had been re-vitrified (Li *et al.*, 2023). It is challenging to elucidate the reasons behind these differences as biopsy introduces an additional bias when assessing the impact of cryopreservation alone.

We acknowledge the strengths and limitations associated with our study. Although the cohort is very homogeneous, consisting of embryos that were all vitrified at the same stage within each group and transferred as single blastocysts, it is still a retrospective design. However, we do not see how reasonable it would be to design a prospective study that would purposely cryopreserve embryos twice. What is more, our number of cases in the double vitrification group is the highest among the other eight studies, as the cohort of Zheng *et al*. (2017) is not directly comparable to ours like the study of Kumasako *et al.* (2009). We additionally considered all critical parameters and characteristics in our cohort relevant to the research question. Thus, we report differences in the time interval from oocyte retrieval to ET and blastocyst expansion score at ET between single and double vitrification groups. Double vitrified embryos remained longer in cryostorage, which validates the nature of the cohort; twice vitrified embryos are expected to remain longer in liquid nitrogen before being thawed for transfer. We do not consider this parameter to be influential when it comes to clinical outcomes. Indeed, prolonged cryostorage of embryos does not negatively influence the CPR or LBR (Cimadomo *et al.*, 2022). We also found that the longer time interval from oocyte retrieval to ET did not influence the LBR in our cohort (Supplementary Table S1), suggesting that the chance of success was not affected by a possible successful previous ET within the two years interval. Other pivotal characteristics for the research question such as endometrial thickness and blastocyst quality at the time of freezing and ET are not reported in the majority of the aforementioned studies. We found the endometrial thickness at ET and blastocyst quality before cryopreservation to be similar between the two groups.

In conclusion, we conducted the first thorough retrospective study to address whether vitrification of blastocysts that have been previously vitrified at the zygote stage is a strategy that is equally effective compared with the vitrification of blastocyst that have never previously been vitrified at any other stage. Indeed, we confidently report that single blastocyst transfer after the practice of double vitrification in our study results in clinical pregnancy and LBRs above benchmark values. We encourage laboratory personnel to re-freeze surplus embryos from FET as a strategy to maintain high LBRs in IVF centres where zygote vitrification is heavily practiced. Our study provides a framework for counselling couples regarding their expectations when banking surplus embryos.

## Supplementary Material

hoad037_Supplementary_Data

## Data Availability

The data underlying this article will be shared on reasonable request to the corresponding author.
