# Supplementary material for: Double vitrification and warming does not compromise the chance of live birth after single unbiopsied blastocyst transfer
Source: Hum Reprod Open. 2023 Sep 22;2023(4):hoad037. doi: 10.1093/hropen/hoad037 (PMC10576635; doi:10.1093/hropen/hoad037)
Supplement: hoad037_Supplementary_Data [file hoad037_supplementary_data.docx]

**Supplementary Table S1:** The outcome of live birth is not impacted by the time interval between oocyte retrieval and embryo transfer (ET)

| **Time interval between oocyte retrieval and ET** | **Live birth** | **No live birth** | **p value** |
| --- | --- | --- | --- |
|  | n=119 | n=288 | 0.538 |
| Within 3 months | 38/119 (31.9%) | 73/288 (25.3%) |  |
| Within 4-6 months | 27/119 (22.7%) | 79/288 (27.4%) |  |
| Within 7-12 months | 29/119 (24.3%) | 68/288 (23.6%) |  |
| Within 13- 24 months | 14/119 (11.7%) | 36/288 (12.5%) |  |
| After 2 years | 11/119 (9.2%) | 41/288 (14.2%) |  |
|  |  |  |  |

Data presented as n (%) and analysed using chi-square test
